# Dose-dependent effects of locally produced microbial phytase on caecal microbiota, hepatic insulin-like *growth factor 1* and growth hormone receptor expression, carcass traits, and immune responses in broiler chickens

**DOI:** 10.14202/vetworld.2026.2038-2050

**Published:** 2026-05-16

**Authors:** Abdullahi Isah, Teck Chwen Loh, Hooi Ling Foo, Anjas Asmara Samsudin, Eric Lim Teik Chung

**Affiliations:** 1 Department of Animal Science, Faculty of Agriculture, Universiti Putra Malaysia, Serdang 43400, Selangor, Malaysia; 2 Institute of Tropical Agriculture and Food Security, Universiti Putra Malaysia, Serdang 43400, Selangor, Malaysia; 3 Department of Bioprocess Technology, Faculty of Biotechnology and Biomolecular Sciences, Universiti Putra Malaysia, Serdang 43400, Selangor, Malaysia; 4 Institute of Bioscience, Universiti Putra Malaysia, Serdang 43400, Selangor, Malaysia

**Keywords:** broiler chickens, carcass characteristics, caecal microbiota, growth hormone receptor, hepatic gene expression, immune response, insulin-like growth factor 1, microbial phytase

## Abstract

**Background and Aim:**

Microbial phytase is extensively used in poultry nutrition to improve phosphorus utilization and reduce the anti-nutritional effects of phytic acid. Beyond nutrient digestibility, phytase may influence intestinal microbiota, immune responses, and growth-related molecular pathways in broiler chickens. However, limited information is available regarding the integrated effects of locally produced microbial phytase on caecal microbial populations, hepatic growth-related gene expression, carcass characteristics, and humoral immunity under the same experimental conditions. Therefore, this study evaluated the dose-dependent effects of dietary microbial phytase supplementation on caecal microbiota, hepatic expression of *insulin-like growth factor 1* (*IGF-1*) and *growth hormone receptor* (*GHR*), plasma immunoglobulin concentrations, and carcass characteristics in broiler chickens.

**Materials and Methods:**

A total of 576 day-old Cobb 500 broiler chicks were randomly assigned to six dietary treatments under a completely randomized design. T1 received a basal diet without phytase supplementation, whereas T2–T6 received basal diets supplemented with microbial phytase at 200, 300, 400, 500, and 600 FTU/kg, respectively. Each treatment consisted of eight replicates with 12 birds per replicate. The experiment lasted for 35 days, covering starter, grower, and finisher phases. Caecal microbial populations were determined using selective culture techniques. Plasma immunoglobulin A and immunoglobulin G concentrations were quantified using enzyme-linked immunosorbent assay kits. Hepatic *IGF-1* and *GHR* mRNA expression levels were analyzed using real-time polymerase chain reaction and quantified using the 2^−^ΔΔCt method with Glyceraldehyde-3-Phosphate Dehydrogenase (*GAPDH*) as the reference gene. Carcass characteristics were evaluated at the end of the feeding trial. Data were analyzed using the general linear model procedure at p < 0.05.

**Results:**

Dietary microbial phytase supplementation significantly increased beneficial caecal bacteria, including *Lactobacillus* spp. and *Streptococcus* spp., while reducing *Escherichia coli* populations compared with the control group (p < 0.05). Birds receiving 600 FTU/kg phytase exhibited the greatest beneficial microbial populations and the lowest *E. coli* counts. Plasma immunoglobulin A and immunoglobulin G concentrations increased significantly in phytase-supplemented birds, particularly during the finisher phase (p < 0.05). Hepatic *IGF-1* and *GHR* mRNA expression levels were significantly upregulated in a dose-dependent manner. Carcass weight and carcass yield also improved significantly following phytase supplementation, with superior responses observed at 600 FTU/kg phytase supplementation. Significant linear responses were detected for most microbial, immunological, molecular, and carcass-related parameters.

**Discussion:**

Locally produced microbial phytase demonstrated multifunctional physiological benefits beyond phosphorus release by improving gut microbial balance, humoral immunity, hepatic growth-related gene expression, and carcass performance in broiler chickens. Supplementation at 600 FTU/kg yielded the greatest overall biological responses and may represent an effective nutritional strategy to improve poultry productivity and intestinal health under tropical production conditions.

## INTRODUCTION

Modern poultry farming increasingly emphasizes the use of nutritional feed additives to improve nutrient utilization, growth performance, gut health, and immune competence in broiler chickens. Among these additives, microbial phytase has received substantial attention for its ability to hydrolyze phytic acid, a major antinutritional factor in plant-derived feed ingredients. The inclusion of phytase in poultry diets improves the bioavailability of phosphorus (P), calcium, amino acids, proteins, and carbohydrates while simultaneously reducing phosphorus excretion into the environment [1, 2]. In addition to improving nutrient digestibility, phytase activity may influence intestinal microbial ecology by liberating bound minerals, such as calcium, iron, and zinc, from phytate complexes [3]. These minerals function as cofactors for numerous microbial enzymes and may promote the proliferation of beneficial intestinal microorganisms. Consequently, phytase supplementation may alter microbial competition within the gastrointestinal tract and improve the availability of nutrients required for microbial metabolism and host physiological functions.

Recent studies have suggested that phytase supplementation may exert physiological effects beyond phosphorus release and nutrient digestibility. Transcriptomic and molecular investigations have demonstrated that phytase may influence muscle-related gene expression and metabolic signaling pathways associated with growth regulation [4]. Furthermore, phytase supplementation has been associated with modulation of hepatic growth-related genes, including *the growth hormone receptor* (*GHR*) and *insulin-like growth factor 1* (*IGF-1*), suggesting a potential role in the endocrine regulation of growth and tissue development [5]. In addition to growth-promoting effects, phytase has been implicated in improving immune responses through enhanced B-cell activity and immunoglobulin production [6]. Increasing evidence indicates that phytase serves as a multifunctional feed additive that improves gut health, intestinal integrity, microbial balance, and immune responses in broiler chickens [7, 8]. The hydrolysis of phytic acid increases the availability of essential nutrients and minerals, thereby supporting improved intestinal morphology, microbial stability, and mucosal immunity [9–11].

Despite the growing body of evidence on the nutritional benefits of phytase, several important knowledge gaps remain. Most previous studies primarily focused on growth performance, phosphorus utilization, bone mineralization, and nutrient digestibility, whereas limited information is available regarding the integrated effects of phytase on caecal microbiota, hepatic growth-related gene expression, carcass traits, and humoral immune responses under the same experimental conditions. Furthermore, studies evaluating locally produced microbial phytase preparations remain scarce, particularly in tropical poultry production systems where feed ingredient composition and environmental conditions may differ substantially from those of imported commercial phytase evaluations. In addition, the dose-dependent physiological effects of phytase on beneficial microbial populations, immune modulation, and endocrine-related growth signaling pathways have not been comprehensively elucidated in broiler chickens. Therefore, a more holistic investigation integrating microbiological, molecular, immunological, and carcass-related parameters is required to better understand the broader biological roles of phytase supplementation in poultry nutrition.

Therefore, this study was conducted to evaluate the effects of graded levels of locally produced microbial phytase on caecal microbial populations, hepatic growth-related gene expression, carcass characteristics, and plasma immunoglobulin concentrations (IgA and IgG) in broiler chickens. The study further aimed to determine whether dose-dependent phytase supplementation could enhance beneficial caecal bacteria, improve carcass yield, upregulate hepatic *GHR* and *IGF-1* expression, and strengthen humoral immune responses in broiler chickens. It was hypothesized that increasing dietary phytase concentrations would positively modulate gut microbial populations, stimulate growth-related molecular pathways, and improve immune function and carcass performance in broiler chickens.

## MATERIALS AND METHODS

### Ethical approval

All experimental procedures involving animals were reviewed and approved by the Institutional Animal Care and Use Committee (IACUC), Universiti Putra Malaysia, Serdang, Selangor, Malaysia, under approval number UPM/IACUC/AUP-R027/2023. The experiment was conducted in accordance with institutional guidelines for the care and use of experimental animals and complied with internationally accepted principles for animal welfare in poultry research. Throughout the experimental period, birds were monitored at least twice daily to assess health status, welfare, and signs of distress, including lethargy, respiratory difficulty, severe lameness, or inability to access feed and water. Birds exhibiting severe clinical symptoms, injury, or compromised welfare were humanely euthanized and removed from the experiment as humane endpoints according to institutional animal ethics regulations.

### Study period and location

The experiment was conducted at the Poultry Research Unit, Department of Animal Science, Faculty of Agriculture, Universiti Putra Malaysia, Serdang, Selangor, Malaysia. Laboratory analyses, involving microbiological and molecular assessments, were performed at the respective research laboratories of Universiti Putra Malaysia. The feeding trial was conducted over a 35-day experimental period comprising starter (0–14 days), grower (15–28 days), and finisher (29–35 days) phases.

### Study design

A completely randomized design was used in this study. A total of 576 day-old Cobb 500 broiler chicks were obtained from Lay Hong Berhad Hatchery, Bukit Rotan, Selangor, Malaysia, with an average initial body weight of 44.41 ± 0.62 g. The birds were randomly assigned to six dietary treatment groups in a closed-house production system. Each treatment consisted of eight replicates with 12 birds per replicate pen. The dietary treatments consisted of T1 = control diet without phytase supplementation, T2 = basal diet + phytase (200 FTU/kg), T3 = basal diet + phytase (300 FTU/kg), T4 = basal diet + phytase (400 FTU/kg), T5 = basal diet + phytase (500 FTU/kg), and T6 = basal diet + phytase (600 FTU/kg).

All chicks were vaccinated intraocularly against Newcastle disease (ND) and infectious bronchitis (IB) on days 7 and 21 using the IB-ND live vaccine. Vaccination against infectious bursal disease was administered intraocularly on day 14. Birds were maintained in plastic cage pens measuring 120 cm × 120 cm (1.44 m²) at a stocking density of 10 birds/m². The poultry house was equipped with an automated ventilation system. Environmental temperature was maintained at 33 ± 1°C on day 1 and gradually reduced to approximately 25 ± 1°C from day 15 onward until the end of the experiment. Relative humidity was maintained within a range of 60%–75%.

The experimental diets were formulated to meet the nutrient requirements recommended for Cobb 500 broilers, as outlined in the Cobb Broiler Performance and Nutrition Supplement [12]. Three dietary phases were prepared, namely starter, grower, and finisher diets (Tables 1–3). Starter and grower diets were offered for 14 days each, whereas the finisher diet was provided from day 29 to day 35. The diets were prepared using a stepwise incorporation of premixes to ensure homogeneity and were stored in a cool, dry environment before feeding. Starter diets were provided in mash form, whereas finisher diets were pelleted using a small-scale compression pelletizer without steam conditioning. The mash feed was mechanically compressed through die openings at near-ambient temperature. Since no thermal conditioning was applied, exposure to temperatures associated with enzyme denaturation (≥70°C) was avoided, thereby preserving phytase activity during feed processing.

**Table 1:** Starter diet composition for broiler chickens fed microbial phytase supplements.

Ingredient (%)	T1	T2	T3	T4	T5	T6
Corn	51.80	51.80	51.80	51.80	51.80	51.90
Soybean meal	36.40	36.40	36.40	36.40	36.40	36.40
Wheat pollard	5.12	5.10	5.09	5.08	5.07	4.95
Crude palm oil	1.21	1.21	1.21	1.21	1.21	1.21
L-Lysine	0.27	0.27	0.27	0.27	0.27	0.27
Monodicalcium phosphate	2.47	2.47	2.47	2.47	2.47	2.48
Calcium carbonate	1.36	1.36	1.36	1.36	1.36	1.36
Choline chloride	0.10	0.10	0.10	0.10	0.10	0.10
Salt	0.25	0.25	0.25	0.25	0.25	0.25
Mineral mix*	0.15	0.15	0.15	0.15	0.15	0.15
Vitamin mix$	0.15	0.15	0.15	0.15	0.15	0.15
Antioxidant#	0.10	0.10	0.10	0.10	0.10	0.10
Toxin binder%	0.10	0.10	0.10	0.10	0.10	0.10
Threonine	0.16	0.16	0.16	0.16	0.16	0.16
L-Arginine	0.00	0.00	0.00	0.00	0.00	0.00
DL-Methionine	0.36	0.36	0.36	0.36	0.36	0.36
Phytase	0.00	0.02	0.03	0.04	0.05	0.06
Total	100.00	100.00	100.00	100.00	100.00	100.00
Metabolizable energy for poultry	3050.00	3050.00	3050.00	3050.00	3050.00	3050.00
Protein	21.04	21.04	21.04	21.04	21.04	21.04
Fat	6.27	6.28	6.28	6.29	6.29	6.29
Fiber	4.34	4.33	4.33	4.33	4.33	4.33
Calcium	0.75	0.75	0.75	0.75	0.75	0.75
Total phosphorus	0.73	0.73	0.72	0.72	0.72	0.72
Available phosphorus for poultry	0.38	0.38	0.38	0.38	0.38	0.38
Salt	0.36	0.36	0.36	0.36	0.36	0.36
Arginine	1.17	1.17	1.17	1.17	1.17	1.17
Lysine	1.15	1.15	1.15	1.15	1.15	1.15
SID Lys	1.26	1.26	1.26	1.26	1.26	1.26
SID Met	0.66	0.66	0.66	0.66	0.66	0.66
SID Met+Cys	0.94	0.94	0.94	0.94	0.94	0.94
SID Thr	0.86	0.86	0.86	0.86	0.86	0.86
SID Tryp	0.24	0.24	0.24	0.24	0.24	0.24
SID Arg	1.38	1.38	1.38	1.38	1.38	1.38

**Nutrient**					**Calculated**	**Analyzed**

Metabolizable energy					3050	3035
Crude protein (%)					21.04	20.97
Calcium (%)					0.75	0.73
Total phosphorus (%)					0.72	0.70
Available phosphorus					0.38	0.37

* Mineral mix = Fe (100 mg), Zn (100 mg), I (2 mg), Mn (110 mg), Cu (20 mg), Se (0.2 mg), and Co (0.6 mg).

$Vitamin premix = retinol (2 mg), cholecalciferol (0.03 mg), α-tocopherol (0.02 mg), thiamine (0.83 mg), riboflavin (2 mg), folic acid (0.33 mg), menadione (1.33 mg), cobalamin (0.03 mg), biotin (0.03 mg), pantothenic acid (3.75 mg), niacin (23.3 mg), and pyridoxine (1.33 mg).

#Antioxidant = butylated hydroxyanisole. %Toxin binder = naturally hydrated sodium, calcium, and aluminium silicates. SID Lys = Standardized Ileal Digestible Lysine, SID Met = Standardized Ileal Digestible Methionine, SID Met+Cys = Standardized Ileal Digestible Methionine + Cystine (Cysteine)

SID Thr = Standardized Ileal Digestible Threonine, SID Tryp = Standardized Ileal Digestible Tryptophan, SID Arg = Standardized Ileal Digestible Arginine

**Table 2:** Grower diet composition for broiler chickens fed microbial phytase supplements.

Ingredient (%)	T1	T2	T3	T4	T5	T6
Corn	52.90	52.90	52.90	52.90	52.90	52.90
Soybean meal	29.55	29.57	29.57	29.57	29.58	29.58
Wheat pollard	10.92	10.88	10.87	10.86	10.84	10.83
CPO	2.00	2.00	2.00	2.00	2.00	2.00
L-Lysine	0.34	0.34	0.34	0.34	0.34	0.34
Monodicalcium phosphate	1.36	1.36	1.36	1.36	1.36	1.36
Calcium carbonate	1.40	1.40	1.40	1.40	1.40	1.40
Choline chloride	0.10	0.10	0.10	0.10	0.10	0.10
Salt	0.35	0.35	0.35	0.35	0.35	0.35
Mineral mix*	0.15	0.15	0.15	0.15	0.15	0.15
Vitamin mix$	0.15	0.15	0.15	0.15	0.15	0.15
Antioxidant#	0.10	0.10	0.10	0.10	0.10	0.10
Toxin binder%	0.10	0.10	0.10	0.10	0.10	0.10
Threonine	0.16	0.16	0.16	0.16	0.16	0.16
L-Arginine	0.07	0.07	0.07	0.07	0.07	0.07
DL-Methionine	0.35	0.35	0.35	0.35	0.35	0.35
Phytase	0.00	0.02	0.03	0.04	0.05	0.06
Total	100.00	100.00	100.00	100.00	100.00	100.00
Metabolizable energy for poultry	2956.00	2956.00	2956.00	2956.00	2956.00	2956.00
Protein	19.00	19.00	19.00	19.00	19.00	19.00
Fat	4.16	4.16	4.16	4.16	4.16	4.16
Fiber	4.23	4.23	4.23	4.23	4.23	4.23
Calcium	0.81	0.81	0.81	0.81	0.81	0.81
Total phosphorus	0.75	0.75	0.75	0.75	0.75	0.75
Available phosphorus for poultry	0.40	0.40	0.40	0.40	0.40	0.40
Salt	0.36	0.36	0.36	0.36	0.36	0.36
Arginine	1.26	1.26	1.26	1.26	1.26	1.26
Lysine	1.27	1.27	1.27	1.27	1.27	1.27
SID Lys	1.16	1.16	1.16	1.16	1.16	1.16
SID Met	0.62	0.62	0.62	0.62	0.62	0.62
SID Met+Cys	0.88	0.88	0.88	0.88	0.88	0.88
SID Thr	0.79	0.79	0.79	0.79	0.79	0.79
SID Tryp	0.21	0.21	0.21	0.21	0.21	0.21
SID Arg	1.29	1.29	1.29	1.29	1.29	1.29

**Nutrient**					**Calculated**	**Analyzed**

ME					2955	2935
Crude protein (%)					19.00	18.97
Calcium (%)					0.81	0.78
Total phosphorus (%)					0.75	0.73
Available phosphorus					0.40	0.39

* Mineral mix = Fe (100 mg), Zn (100 mg), I (2 mg), Mn (110 mg), Cu (20 mg), Se (0.2 mg), and Co (0.6 mg).

$Vitamin premix = retinol (2 mg), cholecalciferol (0.03 mg), α-tocopherol (0.02 mg), thiamine (0.83 mg), riboflavin (2 mg), folic acid (0.33 mg), menadione (1.33 mg), cobalamin (0.03 mg), biotin (0.03 mg), pantothenic acid (3.75 mg), niacin (23.3 mg), and pyridoxine (1.33 mg).

#Antioxidant = butylated hydroxyanisole. %Toxin binder = naturally hydrated sodium, calcium, and aluminium silicates. SID Lys = Standardized Ileal Digestible Lysine, SID Met = Standardized Ileal Digestible Methionine, SID Met+Cys = Standardized Ileal Digestible Methionine + Cystine (Cysteine), SID Thr = Standardized Ileal Digestible Threonine, SID Tryp = Standardized Ileal Digestible Tryptophan, SID Arg = Standardized Ileal Digestible Arginine

**Table 3:** Finisher diet composition for broiler chickens fed microbial phytase supplements.

Ingredient (%)	T1	T2	T3	T4	T5	T6
Corn	48.41	48.41	48.41	48.41	48.41	48.41
Soybean meal	26.75	26.75	26.75	26.75	26.75	26.75
Wheat pollard	16.26	16.23	16.22	16.20	16.19	16.18
Crude palm oil	4.35	4.36	4.36	4.37	4.37	4.37
L-Lysine	0.28	0.28	0.28	0.28	0.28	0.28
Monodicalcium phosphate	1.15	1.15	1.15	1.15	1.15	1.15
Calcium carbonate	1.33	1.33	1.33	1.33	1.33	1.33
Choline chloride	0.10	0.10	0.10	0.10	0.10	0.10
Salt	0.35	0.35	0.35	0.35	0.35	0.35
Mineral mix*	0.15	0.15	0.15	0.15	0.15	0.15
Vitamin mix$	0.15	0.15	0.15	0.15	0.15	0.15
Antioxidant#	0.10	0.10	0.10	0.10	0.10	0.10
Toxin binder%	0.10	0.10	0.10	0.10	0.10	0.10
Threonine	0.11	0.11	0.11	0.11	0.11	0.11
L-Arginine	0.09	0.09	0.09	0.09	0.09	0.09
DL-Methionine	0.32	0.32	0.32	0.32	0.32	0.32
Phytase	0.00	0.02	0.03	0.04	0.05	0.06
Total	100.00	100.00	100.00	100.00	100.00	100.00
ME for poultry	3050.00	3050.00	3050.00	3050.00	3050.00	3050.00
Protein	18.02	18.02	18.02	18.01	18.01	18.01
Fat	6.27	6.28	6.28	6.29	6.29	6.29
Fiber	4.34	4.33	4.33	4.33	4.33	4.33
Calcium	0.75	0.75	0.75	0.75	0.75	0.75
Total phosphorus	0.73	0.73	0.72	0.72	0.72	0.72
Available phosphorus for poultry	0.38	0.38	0.38	0.38	0.38	0.38
Salt	0.36	0.36	0.36	0.36	0.36	0.36
Arginine	1.17	1.17	1.17	1.17	1.17	1.17
Lysine	1.15	1.15	1.15	1.15	1.15	1.15
SID Lys	1.06	1.06	1.06	1.06	1.06	1.06
SID Met	0.57	0.57	0.57	0.57	0.57	0.57
SID Met+Cys	0.83	0.83	0.83	0.83	0.83	0.83
SID Thr	0.70	0.70	0.70	0.70	0.70	0.70
SID Tryp	0.21	0.21	0.21	0.21	0.21	0.21
SID Arg	1.25	1.25	1.25	1.25	1.25	1.25

**Nutrient**					**Calculated**	**Analyzed**

Metabolizable energy					3050	3030
Crude protein (%)					18.10	18.00
Calcium (%)					0.75	0.73
Total phosphorus (%)					0.72	0.70
Available phosphorus					0.38	0.37

* Mineral mix = Fe (100 mg), Zn (100 mg), I (2 mg), Mn (110 mg), Cu (20 mg), Se (0.2 mg), and Co (0.6 mg). $Vitamin premix = retinol (2 mg), cholecalciferol (0.03 mg), α-tocopherol (0.02 mg), thiamine (0.83 mg), riboflavin (2 mg), folic acid (0.33 mg), menadione (1.33 mg), cobalamin (0.03 mg), biotin (0.03 mg), pantothenic acid (3.75 mg), niacin (23.3 mg), and pyridoxine (1.33 mg). #Antioxidant = butylated hydroxyanisole. %Toxin binder = naturally hydrated sodium, calcium, and aluminum silicates. SID Lys = Standardized Ileal Digestible Lysine, SID Met = Standardized Ileal Digestible Methionine, SID Met+Cys = Standardized Ileal Digestible Methionine + Cystine (Cysteine). SID Thr = Standardized Ileal Digestible Threonine, SID Tryp = Standardized Ileal Digestible Tryptophan, SID Arg = Standardized Ileal Digestible Arginine.

No reductions were made to inorganic phosphorus sources in phytase-supplemented diets; therefore, all diets contained similar total phosphorus concentrations. Crude protein, calcium, and total phosphorus concentrations were determined using standard analytical procedures [13], whereas metabolizable energy and phytate phosphorus values were calculated based on ingredient composition. The replicate pen was considered the experimental unit for all statistical analyses.

### Microbial phytase source

The microbial phytase used in this study was supplied by Asian Nutri-chemical (M) Berhad, Serdang, Selangor, Malaysia. The phytase preparation consisted of bacterial 6-phytase intended for use in poultry feed. The locally produced phytase was selected because of its potential cost-effectiveness and compatibility with Malaysian feed ingredients compared with imported commercial phytase products.

Phytase activity in finisher diets was analyzed after feed processing using the standard colorimetric procedure described previously [14]. Feed samples were collected from each dietary treatment after mixing and pelleting procedures. The analyzed phytase activities were 193, 285, 388, 486, and 587 FTU/kg for diets formulated to contain 200, 300, 400, 500, and 600 FTU/kg phytase, respectively, indicating minimal variation from the targeted inclusion rates.

### Caecal digesta microbial count

Post-slaughter caecal samples were aseptically collected from three birds per replicate at weeks 2 and 5 as previously described [15]. The samples obtained from birds within the same replicate were pooled before analysis. Caecal digesta samples were diluted in sterile 0.1% peptone water at a ratio of 1:10, followed by serial dilution from 10^−1^ to 10^−6^. The diluted samples were maintained at 25°C for 1 h before microbial analysis.

The procedure for enumeration of enterobacteriaceae (ENT) and lactic acid bacteria followed previously described methods [16]. Aliquots (100 μL) from the final serial dilutions were plated onto selective media. Lactic acid bacteria were incubated anaerobically at 30°C for 48 h, whereas ENT were incubated aerobically at 37°C for 24 h on eosin methylene blue (EMB) agar.

*Lactobacillus* spp. were enumerated using de Man, Rogosa, and Sharpe agar (Oxoid, Hampshire, UK) and incubated anaerobically at 37°C for 48 h using anaerobic jars with GasPak systems. *Streptococcus* spp. were cultured on M17 agar (Merck, Darmstadt, Germany) and incubated aerobically at 37°C for 24–48 h. *Escherichia coli* populations were enumerated on EMB agar by counting colonies with a characteristic metallic green sheen. Representative colonies were subjected to Gram staining and microscopic examination to confirm media selectivity. *Lactobacillus* isolates were identified as Gram-positive non-spore-forming rods, whereas *Streptococcus* isolates were identified as Gram-positive cocci arranged in chains.

Microbial counts were expressed as log_10_ colony-forming units per gram (log CFU/g) of digesta using the following equation:


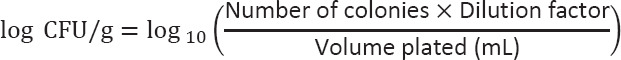


### Plasma immunoglobulin concentration

At weeks 2 and 5, eight birds from each treatment were randomly selected and slaughtered by neck decapitation during the morning period for blood collection. Approximately 5 mL of blood was collected into vacutainer tubes containing ethylenediaminetetraacetic acid and maintained on ice until centrifugation at 3,000 rpm (≈1,000 × *g*) for 15 min at 4°C. Plasma samples were separated and stored at −80°C until analysis.

Plasma concentrations of IgA and IgG were quantified using Chicken IgA ELISA Kit (#4010) and Chicken IgG ELISA Kit (#4020) obtained from Alpha Diagnostic International, San Antonio, TX, USA. Standard concentration ranges were 0–500 µg/mL for IgA and 0–300 µg/mL for IgG. Absorbance was measured at 450 nm using a BioTek ELx800 microplate reader (BioTek Instruments Inc., Winooski, VT, USA). Antibody concentrations were calculated from standard curves after subtraction of blank absorbance values.

Quality control procedures included duplicate measurements for all samples, with intra-assay and inter-assay coefficients of variation maintained below 10%. Standard curves were generated using four-parameter logistic regression in GraphPad Prism software (GraphPad Software, Boston, MA, USA). Relative gene expression was calculated using the 2^-ΔΔCt method [17] with *GAPDH* as the reference gene.

Total RNA isolation and RT-PCR analysis of hepatic *IGF-1* and *GHR*

Tissue collection, RNA extraction, and purification: Immediately after slaughter, liver tissues were collected and freeze-dried. Approximately 30 mg of liver tissue was homogenized with 600 μL of buffer RLT and centrifuged to obtain the supernatant. RNA purity was evaluated using a NanoDrop spectrophotometer (NanoDrop Technologies, Wilmington, DE, USA), and only samples with OD260/280 ratios greater than 1.8 were used for further analyses.

Complementary DNA synthesis: Reverse transcription of 1 μg purified total RNA was performed using the QuantiTect® Reverse Transcription Kit (Qiagen, Hilden, Germany) according to the manufacturer’s instructions. Purified RNA samples were reverse transcribed into complementary DNA.

Real-time polymerase chain reaction (RT-PCR): RT-PCR was performed using a Bio-Rad CFX96 Real-Time PCR System (Bio-Rad Laboratories, Hercules, CA, USA). The reaction mixture consisted of iTaq Universal SYBR Green Supermix (Bio-Rad Laboratories) in a total reaction volume of 20 μL containing 0.4 μM each of forward and reverse primers. Primer sequences and amplicon information are presented in Table 4.

**Table 4:** Primer sequences of *GHR*, *IGF-1*, and *GAPDH* genes used for real-time polymerase chain reaction.

Target gene	Primer sequence (5′→3′)	Product size (bp)	Accession No.	Annealing temperature (°C)
*GHR*	F-AACACAGATACCCAACAGCC	145	NM_001001293.1	54
	R-AGAAGTCAGTGTTTGTCAGGG			
*IGF-1*	F-CACCTAAATCTGCACGCT	140	NM_001004384.2	55
	R-CTTGTGGATGGCATGATCT			
*GAPDH*	F-CTGGCAAAGTCCAAGTGGTG	275	NM_204305.1	53
	R-AGCACCACCCTTCAGATGAG			

bp = base pair, F = forward primer, R = reverse primer.

Amplification conditions consisted of reverse transcription at 50°C for 30 min, initial activation at 95°C for 15 min, followed by 40 amplification cycles involving denaturation at 94°C for 30 s, annealing at 52°C–57°C for 30 s, and extension at 72°C for 1 min. Amplification specificity was confirmed using melting curve analysis. Primer efficiency was evaluated using serial dilutions of complementary DNA.

Relative gene expression of *IGF-1* and *GHR* was calculated using the 2^−^ΔΔCt method [17] with *GAPDH* as the reference gene. Quantification cycle (Cq) values and fold changes were analyzed using CFX Manager software (Bio-Rad Laboratories, USA), and fold changes were calculated using the 2^−ΔΔCt algorithm. All qPCR reactions were performed in technical duplicates.

### Carcass characteristics

At the end of the feeding trial, two birds from each replicate were randomly selected and fasted for 8 h with free access to water before slaughter, in accordance with humane procedures. Carcass yield and cut-part proportions, including breast, thigh, and drumstick, were expressed relative to live body weight using the following equation:


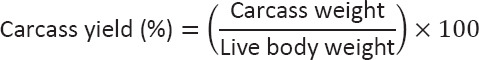


### Statistical analysis

The experiment was conducted using a completely randomized design. Data were analyzed using the general linear model of the Statistical Analysis System software [18]. Differences among treatment means were determined using Duncan’s multiple range test at p < 0.05. Linear and quadratic responses to increasing phytase supplementation were evaluated using orthogonal polynomial contrasts.

The replicate pen served as the experimental unit for growth performance parameters, whereas individual birds were considered the experimental unit for plasma immunoglobulin concentrations and gene expression analyses. Sample size determination was conducted using GPower 3.1 [19] with 80% statistical power to detect a 10% difference in serum IgG concentrations at α = 0.05. Residual normality was verified using the Shapiro–Wilk test, whereas homogeneity of variance was assessed using residual plots. Exact p-values were reported for all significant effects and statistical trends.

## RESULTS

### Caecal microbial count

Birds supplemented with microbial phytase showed greater concentrations of beneficial lactic acid bacteria, including *Lactobacillus* spp. and *Streptococcus* spp., compared with the control group (p < 0.001) (Table 5). In contrast, non-beneficial *E. coli* populations were significantly greater in birds fed the control diet (p < 0.05). The populations of *Lactobacillus* spp. and *Streptococcus* spp. were significantly higher in T6 compared with other phytase-supplemented treatments (p < 0.001).

**Table 5:** Caecal microbial population in broiler chickens fed different levels of microbial phytase.

Parameter (log CFU/g)	T1	T2	T3	T4	T5	T6	SEM	p-value	Linear^1^	Quadratic^2^
*Lactobacillus* spp.	9.27ᵉ	9.28ᵉ	9.35ᵈ	9.44ᶜ	9.53ᵇ	9.68ᵃ	0.0003	<0.0001	<0.0001	<0.0001
*Streptococcus* spp.	5.61ᶠ	5.79ᵈ	5.85ᵈ	6.43ᶜ	7.18ᵇ	7.48ᵃ	0.0310	<0.0001	<0.0001	<0.0001
*Escherichia coli*	7.86ᵃ	7.50ᵇ	7.23ᶜ	6.86ᵈ	6.65ᵈ	6.37ᶠ	0.04	<0.0001	<0.0001	<0.0001

^a⁻f^Means ± SEM within the same row with different superscripts differ significantly at p < 0.05.

1 Linear response calculated using orthogonal polynomial contrasts.

2 Quadratic response calculated using orthogonal polynomial contrasts.

The counts of *Lactobacillus* spp. and *Streptococcus* spp. increased linearly with increasing dietary phytase concentrations (p < 0.001), whereas *E. coli* counts decreased linearly as phytase inclusion increased. In addition, significant quadratic responses (p < 0.001) were observed, indicating a greater microbial modulation effect at higher phytase supplementation levels.

### Plasma immunoglobulin concentration

The humoral immune status of broiler chickens, as determined by plasma IgA and IgG concentrations, is presented in Table 6. Dietary supplementation with microbial phytase significantly increased plasma IgA and IgG concentrations during both the starter (2 weeks) and finisher (5 weeks) phases (*p* < 0.01). Birds receiving T6 exhibited the highest plasma IgA and IgG concentrations among all treatment groups (*p* < 0.05).

**Table 6:** Plasma IgA and IgG concentrations in broiler chickens fed different levels of microbial phytase.

Parameter	T1	T2	T3	T4	T5	T6	SEM	p-value	Linear^1^	Quadratic^2^
**Starter (2 weeks)**										
IgA	23.75ᶜ	29.58ᵇᶜ	30.21ᵇᶜ	32.29ᵇ	34.38ᵃᵇ	39.38ᵃ	1.1834	0.0015	0.0003	0.0913
IgG	47.50	47.50	49.17	52.08	59.17	62.50	2.4757	0.3650	0.0322	0.4917
**Finisher (5 weeks)**										
IgA	26.67ᶜ	35.83ᵇ	38.75ᵃᵇ	39.17ᵃᵇ	42.08ᵃᵇ	43.54ᵃ	1.2037	<0.0001	<0.0001	0.0460
IgG	56.25ᵇ	61.25ᵇ	61.25ᵇ	63.75ᵇ	70.42ᵃᵇ	80.00ᵃ	2.1451	0.0122	0.0009	0.0631

ᵃ⁻ᶜMeans ± SEM within the same row with different superscripts differ significantly at p < 0.05.

IgA = immunoglobulin A, IgG = immunoglobulin G.

1 Linear response estimated using orthogonal polynomial contrasts.

2 Quadratic response estimated using orthogonal polynomial contrasts.

Significant differences in plasma IgA concentrations were observed during both the starter and finisher phases (p < 0.05), whereas plasma IgG concentrations differed significantly among treatments during the finisher phase (p < 0.01). Significant linear increases (p < 0.001) and quadratic responses (p = 0.046) were observed for plasma IgA concentrations, indicating progressive increases with a slight plateau at higher phytase inclusion levels. Plasma IgA and IgG concentrations exhibited significant linear responses during both growth phases (p < 0.001), whereas non-significant quadratic trends, such as starter-phase IgA (p = 0.0913), were considered tendencies.

### Hepatic *IGF-1* and GHR mRNA expression

**Figure 1:**
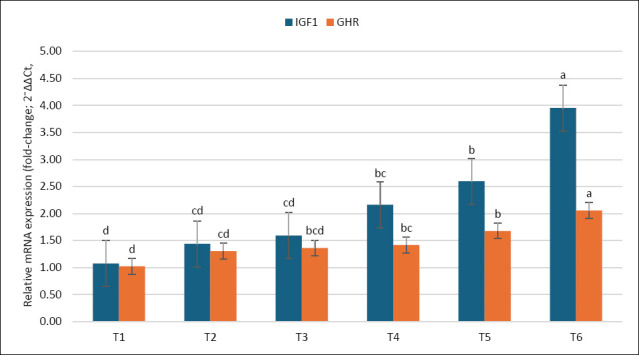
Relative hepatic mRNA expression of insulin-like *growth factor I* (*IGF-1*) and *growth hormone receptor* (*GHR*) in broiler chickens fed graded levels of microbial phytase during the finisher phase. Relative hepatic *IGF-1* and *GHR* mRNA expression levels were quantified using the 2^−^ΔΔCt method and expressed as fold change relative to the control group (T1 = 1.00). Error bars represent mean ± SEM (n = X). Different superscripts (a–d) above bars indicate significant differences among dietary treatments at p < 0.05.

Figure 1 illustrates the relative hepatic expression of *IGF-1* and *GHR* mRNA in broiler chickens fed graded levels of microbial phytase during the finisher phase. Birds supplemented with microbial phytase exhibited significantly greater hepatic gene expression compared with the control group (p < 0.05). The expression of both *IGF-1* and *GHR* increased linearly with increasing dietary phytase levels.

The control group showed the lowest relative expression of both genes, whereas birds fed T5 and T6 exhibited the highest expression levels. Hepatic *IGF-1* expression showed a pronounced dose-dependent upregulation, reaching approximately 3.9-fold higher levels in birds receiving the highest phytase dose compared with the control group. In contrast, *GHR* expression showed a comparatively moderate increase, reaching approximately 2.0-fold in T6 birds.

### Carcass characteristics

Dietary microbial phytase supplementation significantly improved carcass yield compared with the control treatment (p < 0.05) (Table 7). Birds supplemented with 600 FTU/kg phytase exhibited the highest dressing percentage and carcass yield among all dietary treatments. Carcass weight was also significantly enhanced in phytase-supplemented birds.

**Table 7:** Characteristics of internal organs and carcasses in broiler chickens fed microbial phytase supplements.

Parameters	T1	T2	T3	T4	T5	T6	SEM	p-value	Linear^1^	Quadratic^2^
Carcass weight (g)	1852.01ᵈ	1892.31ᶜ	1892.00ᶜ	1909.22ᵃᶜ	1936.01ᵃᵇ	1952.80ᵃ	6.9946	<0.0001	<0.0001	0.9404
Carcass yield (%)	74.23ᶜ	75.16ᵇ	75.17ᵇ	75.25ᵃᵇ	75.35ᵃᵇ	75.57ᵃ	0.0742	<0.0001	<0.0001	0.0026
Breast (%)	29.27	29.69	30.00	30.10	30.27	30.35	0.2589	0.8597	0.1943	0.7087
Leg (%)	18.93	19.67	19.73	19.97	20.02	20.46	0.1923	0.3295	0.0639	0.4961
Thigh (%)	10.46	9.93	10.67	10.93	11.04	11.29	0.1732	0.2656	0.0316	0.7265
Drumstick (%)	8.98	9.00	9.01	9.04	9.18	9.28	0.0992	0.9503	0.3415	0.6795
Back (%)	19.97	19.97	20.26	20.59	20.87	20.96	0.1772	0.4252	0.0338	0.9179
Shank (%)	3.45	3.61	3.61	3.70	3.80	3.85	0.0972	0.0704	0.0105	0.3894
Wing (%)	7.15	7.22	7.22	7.43	7.84	8.03	0.2999	0.2223	0.3101	0.5301
Intestine (%)	3.99ᵃ	3.76ᵃᵇ	4.03ᵃ	3.44ᵇ	3.55ᵃᵇ	3.46ᵇ	0.1542	0.0268	0.0050	0.9537
Heart (%)	0.40	0.47	0.44	0.40	0.41	0.45	0.0244	0.4027	0.3870	0.3482
Spleen (%)	0.11ᵃᵇ	0.07ᵇ	0.16ᵃ	0.12ᵃᵇ	0.07ᵇ	0.08ᵇ	0.0168	0.0037	0.1735	0.0419
Liver (%)	1.61ᵇ	1.50ᵇ	1.86ᵃᵇ	1.80ᵃᵇ	1.97ᵃ	1.84ᵃᵇ	0.1127	0.0567	0.0110	0.4371
Gizzard (%)	2.32	2.33	2.47	2.20	2.33	2.18	0.1540	0.7770	0.4534	0.5369
Abdominal fat (%)	0.64	0.62	0.50	0.53	0.45	0.51	0.0576	0.1630	0.0205	0.3135

ᵃ⁻ᵈMeans ± SEM within the same row with different superscripts differ significantly at p < 0.05.

1 Linear response estimated using orthogonal polynomial contrasts.

2 Quadratic response estimated using orthogonal polynomial contrasts.

T1 = control diet (basal diet without phytase enzyme), T2 = basal diet + phytase (200 FTU/kg), T3 = basal diet + phytase (300 FTU/kg), T4 = basal diet + phytase (400 FTU/kg), T5 = basal diet + phytase (500 FTU/kg), and T6 = basal diet + phytase (600 FTU/kg).

Although most cut-part proportions, including breast, thigh, drumstick, wing, and back percentages, were not significantly affected by dietary treatments, several visceral organ parameters differed significantly among treatments. Significant variations were observed in intestinal, spleen, and liver proportions, indicating that phytase supplementation influenced selected internal organ characteristics in broiler chickens.

## DISCUSSION

### Caecal microbial count

The greater populations of beneficial bacteria, including *Lactobacillus* spp. and *Streptococcus* spp., and the reduced *E. coli* counts observed in birds fed phytase-supplemented diets may be attributed to the ability of phytase to modulate gut microbial ecology, improve nutrient substrate availability, and reduce caecal pH, thereby favoring beneficial microorganisms while suppressing pathogenic bacteria [20, 21]. These findings are consistent with previous reports demonstrating that phytase supplementation reduced harmful bacterial populations such as *E. coli* while promoting beneficial microorganisms, including *Lactobacillus* spp. within the intestinal microbiota [7, 22].

Lactic acid bacteria such as *Lactobacillus* spp. and *Streptococcus* spp. produce lactic acid and hydrogen peroxide, creating an acidic intestinal environment that inhibits the proliferation of enteropathogenic microorganisms [23]. Previous investigations have also demonstrated that microbial phytase supplementation enhances beneficial bacterial populations within the caecum by improving phosphorus digestibility and nutrient availability, thereby supporting microbial proliferation and metabolic activity [8, 24, 25]. The linear and quadratic responses observed in the present study further indicate dose-dependent microbial modulation by phytase supplementation, whereby increasing phytase concentrations enhanced beneficial bacterial populations while suppressing pathogenic microorganisms [7, 26].

### Hepatic *IGF-1* and GHR mRNA expression

The present study demonstrated that dietary microbial phytase supplementation significantly increased hepatic *IGF-1* and *GHR* mRNA expression in broiler chickens, which corresponded with improvements in growth-related responses [10, 11]. Enhanced phosphorus utilization resulting from phytase supplementation may have contributed to the upregulation of *IGF-1* and *GHR* expression [27]. Phosphorus plays an essential role in metabolic regulation and cellular growth, and adequate availability is required for efficient *IGF-1* synthesis, cellular proliferation, and tissue development [28].

Improved nutrient utilization associated with phytase supplementation may also influence *GHR* expression by modulating endocrine and metabolic signaling pathways [29]. The greater expression of hepatic *IGF-1* and *GHR* observed in phytase-supplemented birds suggests that phytase may exert physiological effects beyond phosphorus liberation, potentially contributing to enhanced anabolic activity, nutrient metabolism, and growth regulation in broiler chickens.

### Plasma immunoglobulin concentration

The increased plasma IgA and IgG concentrations observed during both starter and finisher phases are consistent with previous reports indicating that phytase supplementation enhances both mucosal and systemic immune responses in broiler chickens [30]. The improved availability of phosphorus, calcium, zinc, and other trace minerals released through phytate hydrolysis may support immune system development, lymphocyte activity, and antibody production [31]. Several studies have also demonstrated that phytase supplementation improves intestinal mucosal integrity and modulates mucosa-associated microbiota, thereby strengthening barrier function and reducing pathogen colonization [32, 33].

The significant linear increases in plasma IgA and IgG concentrations across dietary treatments further highlight the immunomodulatory role of microbial phytase supplementation. The findings suggest that locally produced microbial phytase may provide additional gut health and immune-supporting benefits in broiler chickens beyond its conventional nutritional role [11]. However, potential confounding factors, including dietary variability and genetic differences among birds, may have influenced immune responses and should be considered when interpreting the results.

The elevated immunoglobulin concentrations observed in phytase-supplemented birds may also be linked to interactions between the intestinal microbiota and host immune regulation. Phytase hydrolyzes phytate, reducing its antinutritional effects and thereby improving nutrient availability for both intestinal microorganisms and the host [34]. This process may favor beneficial microbial populations such as *Lactobacillus* spp. [22], which ferment dietary substrates to produce short-chain fatty acids, including acetate, propionate, and butyrate. These metabolites contribute to intestinal immune homeostasis by maintaining epithelial barrier integrity and regulating immune responses [35]. Furthermore, phytate hydrolysis releases minerals, including phosphorus, calcium, and zinc, which are essential for lymphocyte proliferation, immune signaling, and antibody synthesis [36]. Therefore, the improved plasma immunoglobulin concentrations observed in this study may have resulted from the combined effects of enhanced mineral bioavailability and microbiota-derived immunomodulatory metabolites.

### Carcass characteristics

The improvement in carcass yield observed in phytase-supplemented birds may be attributed to enhanced nutrient digestibility, improved mineral utilization, and better intestinal health status. Increased populations of beneficial bacteria such as *Lactobacillus* spp. together with improved phosphorus and calcium availability may have contributed to enhanced protein deposition and muscle accretion. Improved mineral utilization may also support skeletal development and carcass formation, thereby contributing to greater dressing percentages.

Previous studies have similarly reported improvements in growth performance and carcass characteristics of broiler chickens following phytase supplementation, indicating that the effects extend beyond phosphorus liberation alone [37, 38]. The present findings suggest that the beneficial effects of microbial phytase on gut microbial ecology and nutrient utilization may collectively contribute to improved carcass performance in broiler chickens.

## CONCLUSION

The present study demonstrated that dietary supplementation with locally produced microbial phytase exerted significant positive effects on caecal microbial populations, hepatic growth-related gene expression, humoral immune responses, and carcass characteristics in broiler chickens. Birds supplemented with phytase exhibited higher populations of beneficial bacteria, including *Lactobacillus* spp. and *Streptococcus* spp., and reduced *E. coli* counts compared with the control group. Phytase supplementation also significantly increased plasma IgA and IgG concentrations and enhanced hepatic *IGF-1* and *GHR* mRNA expression in a dose-dependent manner. Furthermore, carcass yield and carcass weight improved in phytase-supplemented birds, particularly at higher phytase inclusion levels. Among the evaluated treatments, supplementation with 600 FTU/kg produced the greatest overall improvements in gut microbial balance, immune responses, growth-related molecular markers, and carcass performance.

The findings of this study highlight the broader physiological roles of microbial phytase beyond conventional phosphorus release and nutrient digestibility. The observed improvements in beneficial intestinal microbiota, immune competence, and growth-related gene expression indicate that phytase may serve as a multifunctional feed additive that supports intestinal health, metabolic regulation, and productive performance in broiler chickens. These responses may contribute to improved feed efficiency, enhanced flock health, and reduced reliance on therapeutic interventions in commercial poultry production systems.

A major strength of this study is the integration of microbiological, molecular, immunological, and carcass-related assessments within a single experimental model, thereby providing a more comprehensive evaluation of phytase functionality in broiler nutrition. In addition, the evaluation of a locally produced microbial phytase offers important practical relevance for poultry industries in tropical production environments, where locally manufactured feed additives may provide cost-effective alternatives to imported commercial enzymes.

Nevertheless, several limitations should be acknowledged. The study did not include nutrient-deficient negative control diets with reduced calcium or available phosphorus concentrations; therefore, the precise contribution of phytase to phosphorus release could not be fully quantified. In addition, gene expression analyses were conducted only during the finisher phase, limiting assessment of temporal or phase-specific molecular responses. The experiment also focused exclusively on broiler chickens under controlled conditions, which may limit extrapolation of findings to other poultry species or commercial production environments.

Future studies should investigate the effects of microbial phytase under reduced-phosphorus dietary conditions to better quantify nutrient matrix values and phosphorus release efficiency. Additional research involving transcriptomic, metabolomic, and intestinal barrier analyses would further clarify the molecular mechanisms underlying phytase-mediated improvements in gut health and immune modulation. Long-term evaluations under commercial farming conditions, as well as investigations involving other poultry species and livestock, are also warranted to determine the broader applicability of locally produced microbial phytase preparations.

In conclusion, locally produced microbial phytase demonstrated significant applied value as a functional feed additive in broiler chicken production. Dietary phytase supplementation improved beneficial caecal microbiota, enhanced humoral immune responses, upregulated hepatic growth-related genes, and improved carcass characteristics in a dose-dependent manner. The results support incorporating microbial phytase, particularly at 600 FTU/kg, as an effective nutritional strategy to improve poultry productivity, gut health, and physiological performance, while potentially enhancing sustainability in modern poultry production systems.

## DATA AVAILABILITY

The data generated during the study are included in the manuscript.

The supplementary data can be made available from the corresponding author upon request.

## AUTHORS’ CONTRIBUTIONS

TCL: Conceptualization and research design, development of experimental protocol, supervision of the feeding trial, validation of experimental procedures and approval of the final version for submission. AI: Feeding trial, sample collection, and preparation of the original manuscript draft. HLF: Laboratory analyses and quality control of biochemical and molecular assays. AAS: Statistical analysis and data interpretation. TCL and ELTC: Manuscript review and editing, contribution to study design refinement, supervision of data interpretation, and generation of tables and figures. All authors read and approved the final manuscript.
